# Capturing spontaneous activity in the medial prefrontal cortex using near-infrared spectroscopy and its application to schizophrenia

**DOI:** 10.1038/s41598-019-41739-4

**Published:** 2019-03-27

**Authors:** Fumiharu Hosomi, Masaya Yanagi, Yoshihiro Kawakubo, Noa Tsujii, Satoshi Ozaki, Osamu Shirakawa

**Affiliations:** 1 Department of Neuropsychiatry, Kindai University Faculty of Medicine, Osaka-sayama, Osaka, Japan; 2 Izumigaoka Hospital, Izumi, Osaka, Japan

## Abstract

Near-infrared spectroscopy (NIRS) is an optimal imaging modality used to examine spontaneous brain activity because it can quietly measure blood flow changes with less physical restriction during the resting state. Here, NIRS was used at rest to measure spontaneous activity in the medial prefrontal cortex (mPFC), a main locus of default mode network. Consistent with previous fMRI studies, magnitude of the spontaneous activity in this region declined with increasing age in healthy subjects. The magnitude reduced in the mPFC of patients with schizophrenia. Additionally, in the mPFC of patients with schizophrenia, the spontaneous activity did not show any age-related decline; the activity was already low in younger patients. Further analysis using fractional amplitude of low-frequency fluctuations confirmed the reduction of spontaneous activity in the mPFC of patients with schizophrenia, consistent with the findings of fMRI studies. Our findings demonstrate the ability of NIRS to evaluate the spontaneous activity in the mPFC of patients with schizophrenia, particularly younger patients. Considering the safety and ease of the NIRS measurements, the current NIRS study of the resting-state activity indicates its utility for clinical applications to schizophrenia, which may facilitate chronological assessment of larger cohorts of patients with schizophrenia in further studies.

## Introduction

Resting-state neuroimaging, which depicts spontaneous brain activities, is a promising tool that can be used for the biological assessments in psychiatric diseases^1–3^. This methodology characterises specific functional networks of the brain by capturing simultaneous low-frequency (<0.1 Hz) fluctuations of cerebral blood flow^4,5^. The network, originally identified as a cluster of brain regions with specifically activated cerebral blood flow when at rest, is called the default mode network (DMN)^6^. This network chiefly comprises the medial prefrontal cortex (mPFC), posterior cingulate cortex (PCC)/precuneus, inferior parietal lobule and lateral temporal cortex^7^. The intensity of regional spontaneous activity has been measured using the amplitude of low-frequency fluctuations of blood flow; this is prominent in the midline regions of the DMN, i.e. mPFC and PCC/precuneus, during the resting state^8,9^.

Among psychiatric diseases, patients with schizophrenia are frequently reported to have an impaired DMN^10–12^. DMN deactivation failure has been reported in the mPFC of patients with schizophrenia while performing cognitive tasks, such as semantic priming and memory tasks, using a subtraction method that quantifies task-induced deactivation^13–15^. Alternatively, DMN activity can be examined by analysing activity during extended rest periods. Several studies have used this analysis method to demonstrate that the amplitude of low-frequency fluctuations (ALFF) is reduced in midline structures of the DMN, such as the mPFC and PCC/precuneus, in patients with schizophrenia^16–23^. Thus, these lines of evidence indicate that in patients with schizophrenia, the DMN activity is diminished, particularly in the locus of the mPFC.

To date, informative albeit high-maintenance modalities, such as positron emission tomography (PET) and functional magnetic resonance imaging (fMRI), have been employed to perform resting-state neuroimaging to detect the DMN. To further develop DMN research for its practical application to psychiatric disorders, a more convenient modality to evaluate DMN activity is warranted. Near-infrared spectroscopy (NIRS) is easy to use and a relatively low-cost imaging modality that is considered safer than PET. NIRS is also suitable for resting-state measurements because it is less physically burdensome (relatively insensitive to movement artifacts) and allows for measuring blood flow changes without the inevitable noise produced during fMRI measurements. Previous studies on the simultaneous recordings of NIRS and fMRI have reported that NIRS has a high concurrent validity with fMRI in resting and cognitive paradigms^24–28^. Instead, the detectable blood flow in NIRS is restricted to the cortical surface area^29,30^ because this device can measure up to the points located at a depth of approximately 2 cm below the scalp^31,32^. Nonetheless, a study previously reported that a NIRS channel superficially covering the mPFC could successfully capture low-frequency fluctuations synchronised with those in the DMN delineated by fMRI^24^. Combined with the evidence that the mPFC is the major region of the brain having reduced DMN activity in the pathophysiology of schizophrenia, it is crucial to determine if the activity changes associated with the DMN are captured in the mPFC of schizophrenia using NIRS. Here, using NIRS during the resting state, we measured low-frequency fluctuations in the mPFC of hospitalised male patients with schizophrenia and healthy males to primarily demonstrate the utility of NIRS for evaluating the spontaneous activity in schizophrenia and to determine the significance of the changes in spontaneous activity in disease phenotypes.

## Results

### Correlation between the Δoxy-Hb_peak_ and age in healthy subjects and the Δoxy-Hb_peak_ change in individuals with schizophrenia

**Figure 1:**
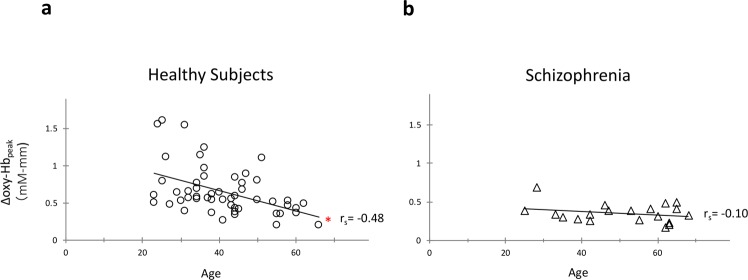
Age-related decline of the Δoxy-Hb_peak_ in mPFC. (**a**) A significant negative correlation was observed between the Δoxy-Hb_peak_ and age in the mPFC of healthy subjects. (**b**) No significant correlation was observed in patients with schizophrenia in the same region.

**Figure 2:**
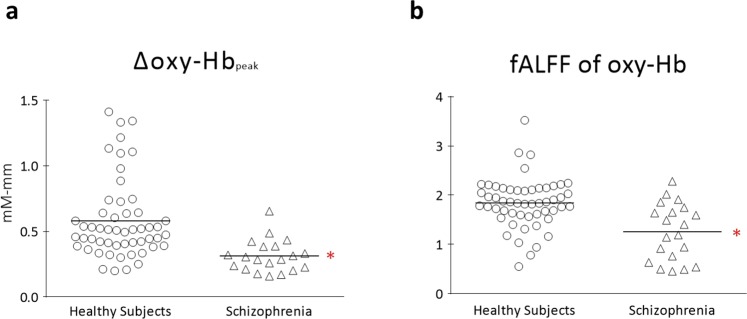
Comparison of the spontaneous activity in oxy-Hb between patients with schizophrenia and healthy subjects in the mPFC. The Δoxy-Hb_peak_ (**a**) and fALFF of oxy-Hb (**b**) significantly decreased in patients with schizophrenia.

Initially, the correlation between the Δoxy-Hb_peak_ and age was examined in the mPFC region of the healthy subjects to investigate the correlation with the findings of previous reports using fMRI. A significant negative correlation was observed between the Δoxy-Hb_peak_ in the mPFC region and age (r_s_ = −0.48, p = 0.0003; Fig. 1a). Next, a general linear model was applied to examine between- group differences between schizophrenia and healthy subjects with age as a covariate. Significant main effects were observed of group (F = 7.53, p = 0.008) and age (F = 10.29, p = 0.002) (Fig. 2a). Although the group–age interaction was not significant, there was a trend towards statistical significance (F = 3.47, p = 0.07). Independent Spearman’s correlation analysis revealed no significant correlations between the Δoxy-Hb_peak_ and age in the schizophrenia group (r_s_ = −0.10, p = 0.67; Fig. 1b). Additionally, no significant correlation was noted between the Δoxy-Hb_peak_ and other clinical variables using the Spearman’s correlation coefficients (p > 0.45).

### Alteration of the fALFF of oxy-Hb in individuals with schizophrenia

Next, fALFF (a proportional measurement that may be dominantly expressed in cortical grey matter with less contamination of physiological noises^9^) was applied to confirm the decrease in spontaneous activity in the schizophrenia group. The general linear model was similarly applied for the fALFF as for the Δoxy-Hb_peak_. A significant main effect was observed of group (F = 15.55, p = 0.0002; Fig. 2b) but not of age (F = 0.002, p = 0.97), and the group–age interaction was not significant (F = 0.51, p = 0.48). Although there were significantly nominal correlations between the fALFF of oxy-Hb and GAF, Delusion and Hallucination scores, no significant difference was observed following the multiple regression analysis (p > 0.23).

### Alteration in deoxy-Hb in schizophrenia

The general linear model for the Δdeoxy-Hb_peak_ showed no significant main effects (group; F = 2.83, p = 0.10 and age; F = 0.44, p = 0.52; Supplementary Fig. 1a) and no significant group–age interaction (F = 0.57, p = 0.45). The general linear model for the fALFF of deoxy-Hb showed a significant main effect of group (F = 9.63, p = 0.003; Supplementary Fig. 1b) but not of age (F = 2.85, p = 0.10), and the group–age interaction was not significant (F = 0.76, p = 0.39). Further, no significant correlation was noted between the fALFF of deoxy-Hb and any clinical variables in Spearman’s correlation analysis (p > 0.21).

## Discussion

The present study quantified spontaneous activity, which was assessed by the magnitude and amplitude of low-frequency fluctuations during the resting state in the mPFC region of healthy subjects and patients with schizophrenia using NIRS. In the mPFC of healthy subjects, the magnitude of spontaneous activity showed an age-related decline, i.e. a negative correlation with age. Previous fMRI studies have shown an age-related decline of DMN, in which age is reported to be negatively correlated with the activity in the frontal node of the DMN^33^. Combined with our results showing age-related decline in the magnitude of spontaneous activity of the mPFC, which was previously reported to be synchronised with the DMN^24^, these lines of evidence indicate that the spontaneous activity of the mPFC captured by NIRS corresponds to a feature of frontal activity in DMN.

Next, we applied NIRS measurement of spontaneous activity to patients with schizophrenia. We focused on hospitalised male patients with apparent social functioning deficits, majority of whom could be probably categorised as psychosis biotype 1 as defined in a previous study^34^, to minimise the clinical variables of the patients to address the heterogeneous nature of schizophrenia. Our results showed that the magnitude of spontaneous activity reduced in the mPFC of patients with schizophrenia. In addition, the spontaneous activity in this region of the patients did not show any age-related decline; their spontaneous activity was low since a young age. Taken together, our results suggest that the reduction of spontaneous activity in the mPFC is more clearly observed in younger patients with schizophrenia, because younger healthy subjects have a larger spontaneous activity. The decrease in spontaneous activity in the patients with schizophrenia was confirmed by the fALFF analysis, which is consistent with the findings of previous resting-state studies which have reported a reduction in the ALFF in the mPFC of patients with schizophrenia^17,18,21–23^. Consistent with our hypothesis, these fMRI studies were accumulated during the first episode of schizophrenia, which generally occurs around adolescence^17,18,21–23^.

**Figure 3:**
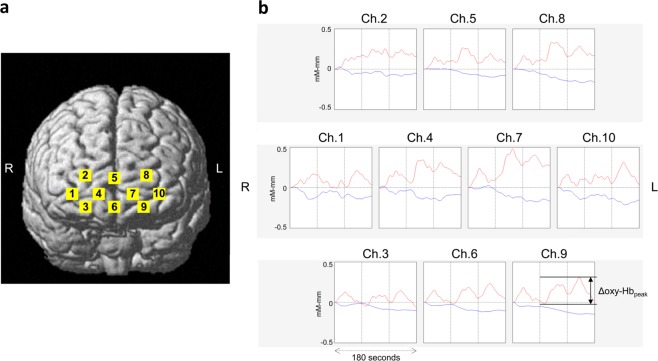
(**a**) Near-infrared spectroscopy (NIRS) channel locations. (**b**) A representative case (a healthy subject) of NIRS measurement during the resting state. Red line, oxy-haemoglobin; blue line, deoxy-haemoglobin.

In this study, an age-related-decline of mPFC activity was detected with the Δoxy-Hb_peak_ but not with other measurements. This inconsistency can be attributed to two factors. One is a technical difference in analysing Δoxy-Hb_peak_ and fALFF. Some consistently activated signals during the resting-state, which was counted in Δoxy-Hb_peak_ analysis, may be removed by excluding very low-frequency fluctuations (<0.01 Hz) in the fALFF analysis. The other reason is a high variability of spontaneous activity in younger healthy subjects as seen in Fig. 1a. The time course of oxy- and deoxy-Hb signals in Fig. 3b demonstrates a potent activation during the resting state, a phenomenon frequently observed in the younger healthy subjects (Fig. 1a). This activation seems to resemble a hemodynamic response, and not just only fluctuations, during the resting state. These observations implied that these activated signals are due to spontaneous thoughts, known as mind wandering^35^, which is likely to occur during the resting state more often in younger individuals^36^. Indeed, the spontaneous activity shown in Fig. 3b was quite similar to the mPFC blood flow observed during mind wandering which was previously reported in an NIRS study^37^.

To the best of our knowledge, this is the first study that demonstrates the utility of NIRS for measuring the spontaneous activity in the mPFC of patients with schizophrenia. The resting-state paradigm has the advantage of assessing patients having some difficulties in adhering to attention-demanding tasks, which is often the case in schizophrenia. Given the safe and convenient clinical applications of NIRS and its suitability for resting-state measurements owing to its comfortability, our results indicate that NIRS measurements at rest may be applied to psychiatry practice concerning schizophrenia. The evaluation of spontaneous activity using NIRS, together with other types of analyses, such as brain functional connectivity^38,39^, in further studies of schizophrenia may provide useful information to better understand changes during the disease process from the first episode itself.

This study also has few limitations. First, NIRS does not technically quantify precise cortical activity. While NIRS channels lack precise annotation to detect a segmentalised cortical area, NIRS signals include contamination of peripheral hemodynamic factors, such as skin perfusion, a major problem in NIRS acquisition^29,40^. Therefore, we cannot completely specify the cortical signals in the NIRS system. However, a previous study that analysed the resting state showed that the radial areas within 3 cm of the NIRS channel on mPFC showed high (>90%) association with cortical signals in fMRI^24^. Second, we cannot exclude the possible influence of antipsychotic medication on spontaneous activity, because all the patients examined in this study were administered antipsychotics. Although no association was observed between the antipsychotic dose and spontaneous activity in the mPFC, the sample size of our cohort is too small to manage the possibility of a statistical type 2 error and to determine the variation caused by antipsychotics. Supplementarily, to examine the influence of antipsychotics on the spontaneous activity of mPFC, we recruited patients with mood or personality disorders who were medicated with antipsychotics (Supplementary Fig. 2). The activity of these patients was not lower than that of healthy subjects, which implies that the reduced spontaneous activity in the patients with schizophrenia was not due to antipsychotic medication. Third, the lack of sufficient sample size may also explain the failure to detect a significant association of the spontaneous activity with the clinical symptoms of the patients with schizophrenia. Lastly, because we examined only male patients in this study, it is unclear whether our results can be generalised to female patients with schizophrenia.

In summary, we applied NIRS to analyse the spontaneous activity associated with the frontal activity of the DMN during the resting state. Accordance was found with previous fMRI findings, whereby the magnitude of spontaneous activity in the mPFC showed an age-related decline in healthy subjects and reduced in patients with schizophrenia compared with age-matched control subjects. In addition, in these patients, the spontaneous activity was found to be low since a young age. Further studies to elucidate chronological changes in the resting-state activity using large cohorts of patients with schizophrenia are warranted; this may be facilitated by applying the current NIRS study into routine psychiatry practice.

## Methods

### Subjects

Fifty-three healthy, right-handed males aged between 23 and 66 years (mean ± s.e.m. = 41.1 ± 1.5) with no history of neurological or psychiatric disorders participated in this study. Twenty right-handed male patients (25–68 years, 50.6 ± 3.0) hospitalised with schizophrenia and having apparent deficits in social functioning based on a GAF score of ≤35 were also included. Two or more psychiatrists diagnosed each patient on the basis of the DSM-5 criteria for schizophrenia. The diagnosis was verified based on detailed clinical observations during hospitalisation. All patients were administered antipsychotics, and none had a history of substance/alcohol abuse. Clinical variables of the patients were as follows: GAF score, 24.2 ± 1.4; illness duration, 27.9 ± 3.5 years; onset age, 23.9 ± 1.6 years; and chlorpromazine-equivalent antipsychotic dose, 804.5 ± 143.7 mg/day. The symptoms of the patients with schizophrenia were assessed based on Clinician-Rated Dimensions of Psychosis Symptom Severity (CRDPSS)^41^ by two research psychiatrists who were blinded to the NIRS data, along with the information obtained from the clinical psychiatrist in charge and ward nurses. The CRDPSS scores were as follows; Hallucinations (mean ± s.e.m. = 2.5 ± 0.3, Delusions (2.6 ± 0.2), Disorganized speech (3.0 ± 0.2), Abnormal psychomotor behaviour (2.5 ± 0.2), Negative symptoms (3.3 ± 0.2), Impaired cognition (3.2 ± 0.2), Depression (0.9 ± 0.1) and Mania (0.3 ± 0.2). A complete description of the study was provided, and written informed consent was obtained from all subjects. The study was approved by the Ethics Committee of the Kindai University Faculty of Medicine and was conducted in accordance with the relevant guidelines and regulations.

### Near-infrared spectroscopy

NIRS measurements were conducted using a 10-channel NIRS device (WOT-100 system; Hitachi High-Technologies Corporation, Tokyo, Japan), as previously described^42–45^. Briefly, the device measured relative changes in oxygenated- (oxy-) and deoxygenated- (deoxy-) haemoglobin (Hb) concentrations using two wavelengths (705 and 830 nm) with a sampling rate of 200 ms. Optical data were analysed using the modified Beer–Lambert law to calculate the signals reflecting changes in Hb levels expressed as arbitrary units (mM–mm) because the differential path lengths of the wavelengths are indefinite in this system^42–45^. The NIRS probe unit has a 2 × 4 alternating arrangement of irradiation and detection positions. The lowest probes were positioned along the Fp1-Fp2 line, according to the International 10–20 system^44,46^. The distance between pairs of emission and detector probes was set at 30 mm, and the measurement area between the probes was defined as a channel. The arrangement of channels covered the entire forehead to monitor activation in prefrontal regions^42,47^. This experimental setup was corroborated by that of a multi-individual study of anatomical craniocerebral correction using the International 10–20 system^46–48^. Three-dimensional coordinates of the channels were obtained from six healthy subjects using a three-dimensional digitiser (Patriot, POLHEMUS, Inc., Colchester, Vermont, USA), and the mean estimate for the spatial registration of the channels using the probabilistic-determination method^46,47^ was mapped onto the Montreal Neurological Institute space based on NIRS-SPM (http://bispl.weebly.com/nirs-spm.html#/), as shown in Fig. 3a and Supplementary Table S1.

### Resting-state paradigm

For the resting-state acquisition, the subjects were seated in a comfortable chair in a silent room. They were instructed to keep their eyes open and remain still while focusing on the central fixation point (cross hair) displayed on a monitor. The NIRS measurement commenced when the cross hair appeared on the monitor and continued for three minutes at rest.

### Data analysis

#### Magnitude of low-frequency fluctuations (Δoxy-Hb_peak_)

Relative changes in oxy-Hb signals were analysed according to a previous report that demonstrated a strong correlation between oxy-Hb NIRS measurements and blood oxygenation level-dependent signals measured using fMRI^49^. Further, relative changes in deoxy-Hb signals were analysed supplementarily. The time courses of oxy- and deoxy-Hb signals were diagrammed using the BRainSuite_Analyzer (BRSystems. Inc., Kanagawa, Japan) with low pass filter (<0.1 Hz) to eliminate physiological noises, such as those of heart beat, respiration and quick body movements^50,51^. Figure 3b presents the representative time course of oxy- and deoxy-Hb signals during the resting state. To represent the magnitude of low-frequency fluctuations during the resting state, Δoxy-Hb_peak_ was indexed as the peak value minus the lowest value for oxy-Hb signals during the resting state (Fig. 3b). The Δoxy-Hb_peak_ was calculated for the 10 individual channels of NIRS, and selectively, the following region of interest (ROI) was chosen to examine the spontaneous activity. The ROI was set as channels 5 and 6 located on the superficial part of the mPFC, and then, the Δoxy-Hb_peak_ of channels 5 and 6 were averaged for the ROI signal.

#### Fractional Amplitude of the low frequency fluctuations (fALFF)

fALFF was calculated using the ALFF/fALFF software (BRSystems Inc.; Brainsuit ALFF; Kanagawa, Japan) according to previous reports^9,52^. In this software, first, the time series for each channel was transformed to a frequency domain using the fast Fourier transform, and the power spectrum was obtained. Then, the square root was calculated at each frequency of the power spectrum. The sum of the square root across the low-frequency range (0.01–0.08 Hz) was divided by that across the entire frequency range (0–0.25 Hz) to calculate fALFF. The fALFF was analysed for ROI as selected in the previous section.

### Statistical analysis

Between-group difference of the NIIRS data between the patients with schizophrenia and healthy subjects was examined using a general linear model with the Δoxy−/deoxy-Hb_peak_ or fALFF as dependent variables, group (patients with schizophrenia/healthy subjects) as a between-subject factor and age as a covariate. To examine the relationships between the Δoxy−/deoxy-Hb_peak_ or fALFF and clinical variables in patients with schizophrenia, Spearman’s correlation coefficient (r_s_) was initially analysed. If any significance was found, multiple regression analysis was conducted with the NIRS measurements as dependent variables, thereby controlling for other potential confounding variables (age and antipsychotic dose), and added factors such as GAF and CRDPSS scores. The threshold for the significance of p-values was set at 0.05. All statistical tests were two-tailed and were performed using GraphPad Prism 6.0 for Windows version 6.07 (GraphPad Software, Inc., La Jolla, CA, USA) or SPSS version 25.0 (IBM Inc., New York, USA).

### Exclusion of probe error channels

Within the 10 channels measured using NIRS, those judged to be probe errors for each subject by the WOT-100 software were excluded from the analysis. According to this criterion, ch. 1 and ch. 9 were excluded for one healthy subject and ch. 10 was excluded for two. In schizophrenia, ch. 2 and ch. 3 were excluded for two patients, ch. 7 and ch. 9 were excluded for one, ch. 8 was excluded for three, and ch. 10 was excluded for seven. Notably, ch. 4, ch. 5 and ch. 6 were intact for all healthy subjects and patients with schizophrenia.

## Supplementary information

Supplementary Information

## Data Availability

The datasets generated and/or analysed during the current study are available from the corresponding author on reasonable request.
