# Supplementary material for: Capturing spontaneous activity in the medial prefrontal cortex using near-infrared spectroscopy and its application to schizophrenia
Source: Sci Rep. 2019 Mar 27;9:5283. doi: 10.1038/s41598-019-41739-4 (PMC6437166; doi:10.1038/s41598-019-41739-4)
Supplement: Supplementary file 1 — Supplementary Information [file 41598_2019_41739_MOESM1_ESM.pdf]

Supplementary Information

Capturing spontaneous activity in the medial prefrontal cortex using near-infrared spectroscopy and its application to schizophrenia

Fumiharu Hosomi[1#], Masaya Yanagi[*1#], Yoshihiro Kawakubo[1], Noa Tsujii[1], Satoshi Ozaki[2] and Osamu Shirakawa[1]

[1]Department of Neuropsychiatry, Kindai University Faculty of Medicine, Osaka-sayama, Osaka, Japan

[2]Izumigaoka Hospital, Izumi, Osaka, Japan

[#] Equal contribution

Table S1. Montreal Neurological Institute (MNI) coordinates of the estimated channel positions

	MNI coordinates		
	x (mm)	y (mm)	z (mm)
Ch. 1	34	67	3
Ch. 2	24	70	17
Ch. 3	24	70	−7
Ch. 4	14	74	4
Ch. 5	2	68	16
Ch. 6	3	70	−7
Ch. 7	−12	74	4
Ch. 8	−21	70	16
Ch. 9	−20	70	−7
Ch. 10	−32	66	3

Ch. = channel

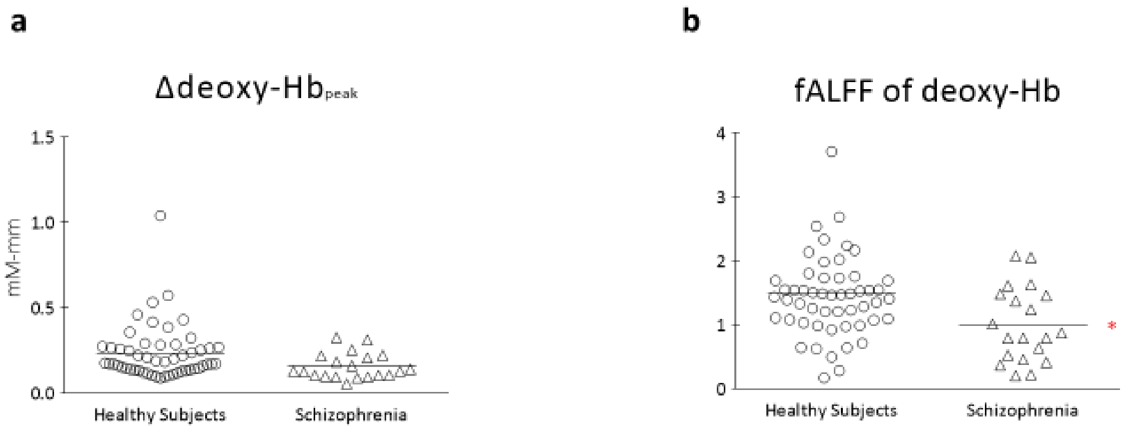

Supplementary Figure 1. Comparison of the spontaneous activity in deoxy-Hb between patients with schizophrenia and healthy subjects in the mPFC. (**a**) Δdeoxy-Hb_{peak}. (**b**) fALFF of deoxy-Hb. The fALFF of deoxy-Hb, but not the Δdeoxy-Hb_{peak}, significantly decreased in patients with schizophrenia.

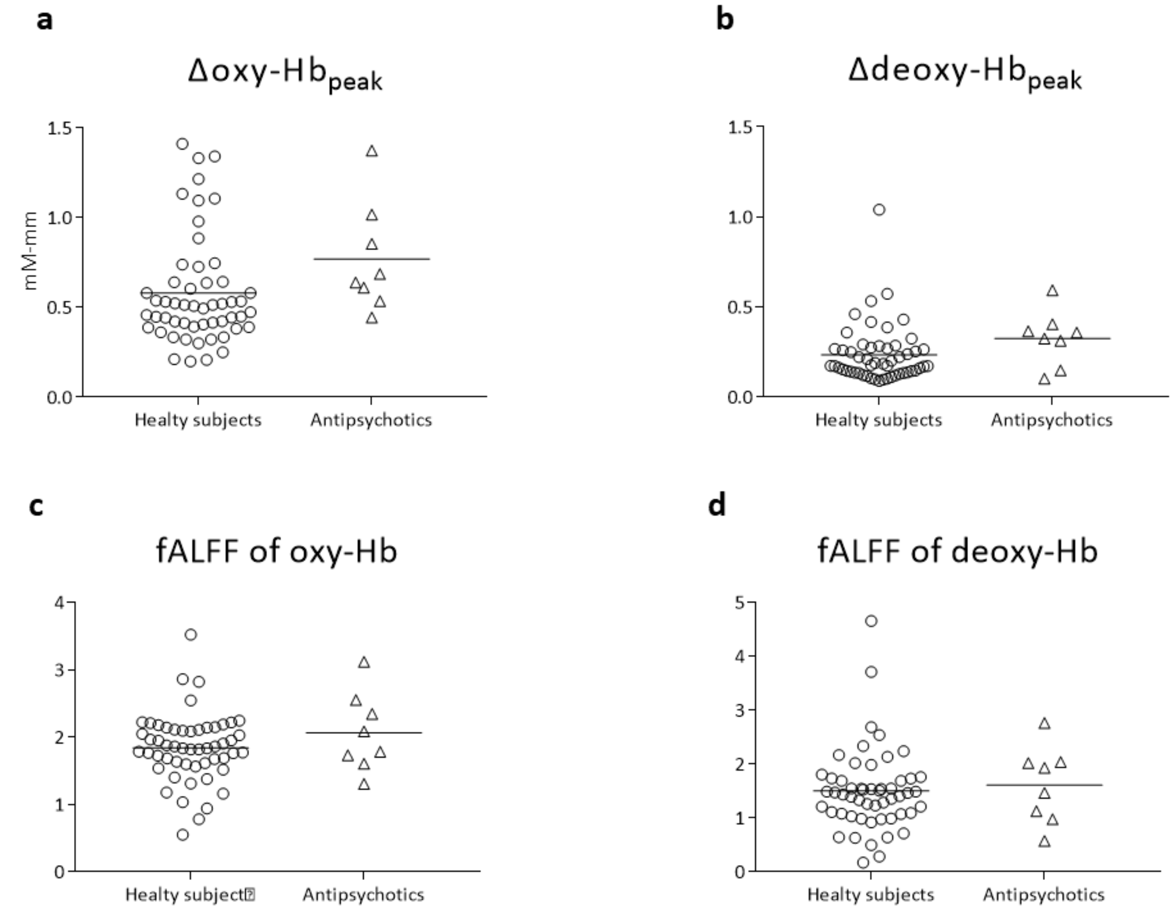

Supplementary Figure 2. To exploratorily examine the influence of antipsychotics on the spontaneous activity, eight male patients (three patients with major depression, four with bipolar disorder, and one with personality disorder; age, 24–60 years, mean age, 40.0 ± 5.4) who were medicated with antipsychotics (chlorpromazine-equivalent antipsychotic dose, 298.0 ± 89.5 mg/day) were recruited for the NIRS measurements. Compared with healthy subjects, they did not show significant differences in any of the parameters (**a.** Δoxy-Hb$_{peak}$. **b.** Δdeoxy-Hb$_{peak}$. **c**. fALFF of oxy-Hb. **d**. fALFF of deoxy-Hb) for the spontaneous activity in the mPFC (the general linear model; $F = 2.72$, $p = 0.11$ for Δoxy-Hb$_{peak}$, $F = 2.22$, $p = 0.14$ for Δdeoxy-Hb$_{peak}$, $F = 1.38$, $p = 0.25$ for fALFF of oxy-Hb, $F = 0.21$, $p = 0.65$ for fALFF of deoxy-Hb).
